# CHALLENGES RELATED TO DATA SOURCES FOR RWE IN AGING RESEARCH

**DOI:** 10.1093/geroni/igac059.771

**Published:** 2022-12-20

**Authors:** Kevin Lu

**Affiliations:** University of South Carolina, Columbia, South Carolina, United States

## Abstract

Numerous challenges remain in research to generate RWE for FDA approvals and other health care decision-makers or stakeholders. This is particularly true for research to evaluate the effectiveness of pharmaceutical care and patient outcomes in older populations. For example, data sources available for RWE in the older populations are limited and data access can be daunting; identifying appropriate data sources for FDAapprovals remain challenging. Most of the FDA approvals are based on RWE rely on multiple data sources instead of a single database. In addition, pharmacoepidemiologic studies in older adults using RWD are often limited in capturing important clinical information and prognostic factors that are useful for decision-makers. Polypharmacy, frailty, and patient outcomes measures are often inconsistent depending on specific databases and thus produce controversial results. We will discuss different data sources appropriate for pharmacoepidemiology research and make comparisons and contrasts of different research approaches using different databases.

